# Clinical Characteristics of 10 Pregnant and Postpartum Women With Extracorporeal Membrane Oxygenation: A Retrospective Study

**DOI:** 10.3389/fmed.2021.778889

**Published:** 2022-01-03

**Authors:** Qiao Gu, Weihua Peng, Ying Zhu, Shaosong Xi, Mengyuan Diao, Wei Hu, Xiaokang Zeng

**Affiliations:** Department of Critical Care Medicine, Affiliated Hangzhou First People's Hospital, Zhejiang University School of Medicine, Hangzhou, China

**Keywords:** extracorporeal membrane oxygenation, pregnant, obstetric, postpartum, critical care, clinical characteristics

## Abstract

**Background:** The aim of study was to summarize the clinical characteristics and experience of extracorporeal membrane oxygenation (ECMO) in pregnant and postpartum patients.

**Methods and Results:** We retrospectively reviewed 131 consecutive ECMO patients at our center from May 2015 to May 2021. A total of 10 Chinese patients were pregnant or postpartum at the time of ECMO initiation. Patients ranged in age from 25 to 36 years (median age 30.5 years). The ECMO duration ranged from 3 to 31 days (median duration 8 days). There was a stabilizing trend of acid-base balance and decreasing lactic acid over the 3 days following ECMO initiation. Seven (70%) patients survived at least 48 h after weaning from ECMO. Four (40%) patients survived until discharge, and four (40%) fetuses survived until discharge.

**Conclusion:** ECMO provides a suitable temporary cardiopulmonary support for pregnant and postpartum patients. ECMO shows a favorable effect on short-term stability in critical obstetric patients.

## Introduction

Extracorporeal membrane oxygenation (ECMO) is an extracorporeal life-support technology for patients suffering from refractory cardiac shock and/or respiratory failure. In ECMO, venous blood is oxygenated and pumped back into the patient's vessels *via* a centrifugal pump located outside the body, in order to meet the body's demands (1–3). To best treat different conditions in patients, venoarterial (VA) and venovenous (VV) are the two basic types of ECMO, and these use different cannulation vessels. In certain centers, some additional, modified types of ECMO are accepted because additional venous or arterial cannulation can help manage several clinical challenges. A jugular venous backflow cannula is commonly added in cases with concomitant severe lung failure on VA-ECMO [i.e., for veno-arterio-venous (VAV) support], whereas venous drainage cannula (VVA) or arterial backflow cannula (VAA) additions are rarely chosen to support the circulation (4).

In recent decades, ECMO has been widely used as an approach for treating critically ill patients, especially infants with congenital diaphragmatic hernia or pneumonia, as well as adults with fulminant myocarditis (5, 6). Clinicians are more likely to initiate an ECMO procedure in these specific populations, given the clear and significant benefits. Previous cardiopulmonary resuscitation (CPR) is also an indication for ECMO use because it allows patients to recovery from refractory cardiac arrest (7).

Pregnant and postpartum women often have a rapid onset of symptoms complicated with underlying disease. Even for experienced physicians, the decision to use ECMO is challenging because a number of unique factors (e.g., the fetus, hypercoagulability, liquid burden) must be taken into consideration separately. These various factors create clinical situations that are intractable with respect to diagnosis and/or management. According to the Extracorporeal Life Support Organization (ELSO), a progressive increase of ECMO use has been seen in recent decades (8). However, published studies involving ECMO use in pregnant and postpartum women are significantly lacking, especially those concerning Asian obstetric patients (9). ECMO data from pregnant and postpartum women are sometimes published together with information from non-obstetric patients. However, there is a great degree of variability in pregnant and postpartum patients, which makes it difficult to evaluate the effectiveness of ECMO treatment when data are not provided separately. Here, we reviewed our clinical records from the most recent 6 years; we report data on ECMO use in pregnant and postpartum women and provide additional characteristics of our study population.

## Materials and Methods

We completed a retrospective review of all patients treated with ECMO who were admitted to our hospital between May 2015 and May 2021; the first pregnant patient treated with ECMO in our hospital was in 2015. A total of 131 ECMO procedures were performed in our center during this time. Of these, 10 patients were pregnant or postpartum women, and all consecutive patients are included in this study. Postpartum patients were only included if ECMO cannulation occurred within 8 weeks of delivery. Data were collected from our institution's electronic medical record system. History of illness, past medical history, laboratory tests, rescue therapies, and discharge status were recorded for all patients.

Our hospital performed its first ECMO procedure in 2009, and has since become a leading ECMO regional center. Our hospital also works as a regional center for critically ill perinatal women and, therefore, our center sees a higher rate of ECMO use in pregnant women. All 10 patients were treated in our general intensive care unit (ICU), regardless of the type of ECMO performed.

Two ECMO systems were on service in our center during the time of this study: (1) the Rotaflow Centrifugal Pump (Maquet Inc, Rastatt, Germany) combined with a Quardox D oxygenator, and (2) the Cardiohelp System (Maquet Inc, Rastatt, Germany). Both systems are commonly used in China. We preferred to use the right femoral vein for drainage cannulation followed by the left femoral vein, because the left femoral vein is more commonly curved. For backflow cannulation, either femoral artery was used for VA-ECMO and either jugular vein was used for VV-ECMO. If an additional cannulation was needed for VAV ECMO, either jugular vein was used. However, decisions were made after considering the bilateral femoral vascular ultrasound.

In our ICU, decisions to initiate ECMO treatment were made by the same ECMO team leader after discussing the state of patient illness in our workgroup, or sometimes more quickly in emergency situations. Since our hospital is a regional center for critically ill perinatal women, patients can be canulated and administered ECMO treatment in local clinical departments, and later transferred to our center for monitoring and further treatments. While a patient was being considered as candidate for ECMO, our ICU colleagues would review patient history and evaluate with ultrasound. Through discussion, our team leader would then decide whether to initiate ECMO and where to perform the cannulations. All 10 ECMO cannulations were completed using the advanced Seldinger puncture technique with ultrasound guidance, and the location of guide wires and drainage cannulations was confirmed.

For circulatory failure, VA-ECMO serves to improve oxygen supply and delivery. The drainage catheter tip was typically placed in the left atrium, and a primary blood flow of 3.5–4.5 L/min was set to maintain a mean arterial pressure of 60–70 mmHg, or sometimes 70–80 mmHg in external cardiopulmonary resuscitation (ECPR) patients to achieve better cerebral perfusion. Blood flow through the aortic valve was frequently monitored with ultrasound doppler because a high VA-ECMO blood flow can lead to an elevated afterload, causing thrombosis in the left ventricle or the left atrium. If circulation improved, adjustment of blood flow was considered in order to keep the aortic valve activated; an intra-aortic balloon pump (IABP) was used as an alternative to decompress the left ventricle. Continuous renal replacement therapy (CRRT) was commonly prioritized to maintain a stable internal environment unless the patient had not suffered any aggravated kidney injury or oliguria.

With regard to VV-ECMO in respiratory failure, low volume protective ventilation and prone position ventilation were typically administered prior to ECMO, and were needed after ECMO initiation. We were extremely careful to ensure that the drainage cannulation tip was positioned in the inferior vena cava to minimize recirculation. Additionally, we adjusted the flow and oxygen concentration of VV-ECMO to improve oxygen supply and remove excess carbon dioxide. In our institution, we aim for 88–95% blood oxygen saturation and 35–45 mmHg PCO_2_. No awake VV-ECMO procedures were performed in this study, and rapid frequency of breath did improve, but a rapid and shallow breath still existed after ECMO initiation. In order to avoid further lung injury caused by elevated driving pressure, we continued to reduce oxygen consumption and protect lung function through sedation and analgesia.

As far, specific ECMO removal suggestions for pregnant patients were rarely described in prior studies while the situations could be complicated. In particular, anticoagulant contraindications and high risk for thrombosis could exist in a same special person. In our center, readiness for weaning from ECMO and bedside echocardiography evaluation were performed daily. Aortic VTI ≥ 10 cm, LVEF > 20–25%, and lateral mitral annulus peak systolic velocity >6 cm/s were recommended in some literatures with non-pregnant patients associated with successful weaning (10, 11). Similar VA-ECMO removal criteria were adopted in our study. Before removal of VV-ECMO, regular trials with the sweep gas turned off were required in our center. Weaning criteria from VV-ECMO based on EOLIA study are: P_a_O_2_ ≥ 60 mmHg, S_a_O2 ≥ 90%, with FiO_2_ ≤ 60%; P_a_CO_2_ ≤ 50 mmHg or PH ≥ 7.36, with respiratory rate ≤ 28/min; P_plat_ ≤ 28 cmH_2_O; and no signs of acute cor-pulmonale (12).

Once-daily fetal monitoring was routinely performed by obstetricians in patients who were still pregnant at the time of ECMO initiation. Intrauterine fetal demise was commonly induced, or fetuses were actively delivered by Cesarean section. In the case of fetal survival, corticosteroids were more likely used first to promote fetal lung maturation and to prepare for unexpected regular uterine contractions. For patients in the early stages of pregnancy, we discussed with the department of obstetrics to assess the need to continue the pregnancy. In the third trimester, an early Cesarean section was the preferred approach to decompress the patient's circulatory and respiratory load.

## Results

In this study, 10 pregnant and postpartum women received ECMO treatment in our center from May 2015 to May 2021; patient characteristics are listed in Table 1. All patients were from China, and relatively few cases have been previously reported in Asian patients. Of the 10 patients in this study, seven (70%) had ECMO initiated at our hospital, of which six (60%) were catheterized in our ICU and one (10%) was transferred to ICU after catheterization in the emergency room. The other three (30%) patients were transferred to our ICU after ECMO catheterization at local hospitals. The patients ranged in age from 25 to 36 years (median age 30.5 years). At admission, two (20%) patients were in the first trimester, five (50%) patients were in the third trimester, and three (30%) patients had delivered or miscarried. ECMO was initiated in three (30%) patients prior to delivery; two (20%) patients did not undergo regular obstetric examination during pregnancy; one (10%) patient was examined for splenic aneurysm and was recommended splenic aneurysm surgery or termination of pregnancy, but resolutely refused these treatments.

**Table 1:** Basic characteristics of patients.

**Patient**	**1**	**2**	**3**	**4**	**5**	**6**	**7**	**8**	**9**	**10**
**ECMO type**	**VA**					**VAV**	**ECPR**		**VV**	
Age (year)	30	31	31	33	28	26	25	31	36	25
BMI (kg/m^2^)	32.04	27.58	N/A	N/A	23.94	28.83	19.53	N/A	27.34	17.58
History of pregnancy	G5P1	G1P0	G2P0	G1P0	G2P1	G2P0	G3P0	G1P0	G5P1	G1P0
GA when admitted to our hospital	33 weeks 3 days	34 weeks 1 day	12 weeks 6 days	32 weeks 1 day	2 days after delivery	39 weeks 2 days	After induced abortion	32 weeks 5 days	54 days after delivery	12 weeks 5 days
GA when admitted to ICU	33 weeks 3 days	0 day after delivery	12 weeks 6 days	32 weeks 1 day	0 day after delivery	39 weeks 2 days	After induced abortion	0 day after delivery	51 days after delivery	12 weeks 5 days
GA when ECMO initiated	2 days after delivery	1 day after delivery	12 weeks 6 days	32 weeks 1 day	2 days after delivery	0 day after delivery	After induced abortion	32 weeks 5 days	54 days after delivery	37 days after delivery
Time of MV before ECMO initiated	0 day	1 day	0 day	0 day	0 day	0 day	0 day	0 day	3 days	38 days
Where ECMO Initiated	ICU Bedside	ICU Bedside	ICU Bedside	Local hospital ER	ICU Bedside	ICU Bedside	Local hospital ER	ER	Local hospital ICU bedside	ICU Bedside
Regular obstetric examination	NO	NO	YES	YES	YES	YES	YES	YES	YES	YES
Fetus when ECMO initiated	C-section, survived	C-section, survived	Normal fetal	Normal fetal	C-section, intrauterine demise	C-section, survived	Induced abortion, intrauterine demise	Intrauterine demise	Stillbirth	Spontaneous abortion

*ECPR, external cardiopulmonary resuscitation; BMI, body mass index; GA, gestational age; MV, mechanical ventilation; ER, emergency room; C-section, Cesarean section*.

After reviewing medical history and treatment process for each patient in the electronic medical record system, we summarized patient information at the time of ECMO initiation in Table 2. Various etiologies in our study were Eisenmenger syndrome, cardiac arrest secondary to induced abortion syndrome, fulminant cardiomyopathy, pulmonary thrombosis, cardiac arrest after hemorrhagic shock, interstitial pneumonia, infection-induced acute respiratory distress syndrome (ARDS), and cardiogenic shock after aortic dissection. Among these, cardiac arrest after induced abortion syndrome and hemorrhagic shock were rarely reported. Patients receiving VA-ECMO all had severe circulatory failure for various reasons. Moreover, both patients receiving VV-ECMO could not maintain oxygen delivery after conventional treatment (e.g., low tidal volume ventilation or prone positioning), and were treated with ECMO as a salvage treatment.

**Table 2:** Information of patient disease.

**Patient**	**Reasons for admission to the ICU**	**Basis disease**	**Conditions at ECMO initiation**	**Other main treatment**
**VA**				
1	Eisenmenger syndrome	Congenital heart disease, PDA, thrombocytopenia	PASP > 150 mmHg, SBP 120 mmHg,PaO_2_/FiO_2_ = 55 with high-dose NE	PGI2, S-G, CRRT, Vasoconstrictor
2	Stanford type A aortic dissection		Circulation failure after Bentall and C-section surgery, increasing lac 17.4 mmol/L, PaO_2_/FiO_2_ = 54.7 with high-dose NE and inotropic drugs, hypoxia	CRRT, IABP, Vasoconstrictor
3	Fulminant carditis, ROSC		Poor heart contractility after CPR, cardiac edema, EF 18%, increasing lac 14.1 mmol/L with high dose inotropic drugs and NE	CRRT, IABP, Vasoconstrictor
4	Circulation failure after ROSC	Pulmonary hypertension detected in obstetric test	Consideration of massive PE, increasing lac 13.8 mmol/L with high-dose NE, hypoxia	CRRT, thrombolytic therapy, Vasoconstrictor
5	Severe metabolic acidosis intrauterine demise	Left femur fracture 3 months ago	Consideration of AFE, thrombocytopenia, certain low-risk PE, lac > 20 mmol/L with high-dose NE and steroid	CRRT, Vasoconstrictor
**VAV**				
6	Eisenmenger syndrome	Congenital heart disease, VSD	PASP > 150 mmHg, SBP 110–120 mmHg, PaO_2_/FiO_2_ = 41 with high-dose NE	PGI2, S-G, Vasoconstrictor
**ECPR**				
7	Induced abortion syndrome	Intrauterine demise, CA	Hardly maintain SBP > 35 mmHg even with NE and epinephrine I.V	Vasoconstrictor
8	Hemorrhage shock, intrauterine demise	Ruptured splenic artery aneurysm, CA	CA upon admission, no ROSC after 30 min, HB 64 g/L after blood transfusion, increasing lac 15.7 mmol/L	CRRT, uterine water bag oppression, Vasoconstrictor
**VV**				
9	Interstitial pneumonia, pneumocystis carinii infection	CADM	Misdiagnosed in local hospital, RP-ILD, PaO_2_/FiO_2_ = 53.1, pneumomediastinum	Prone position, low tidal volume ventilation, RM, NMB
10	Infection-induced ARDS	Cerebral hemorrhage	ARDS, PH 7.16, lac 9.3 mmol/L, PaO_2_/FiO_2_ = 45, sepsis, pneumomediastinum	Prone position, low tidal volume ventilation, RM, NMB

*PASP, pulmonary arterial systolic pressure; NE, norepinephrine; PGI2, prostaglandin-2; S-G, Swan-Ganz catheter; RM, recruitment maneuvers; NMB, neuromuscular blocking agent; ARDS, acute respiratory distress syndrome; CADM, clinical asymptomatic dermatomyositis; RP-ILD, rapidly progressive interstitial pneumonia; CA, cardiac arrest; ROSC, restoration of spontaneous circulation; IV, intravenous; HB, hemoglobin; CRRT, continuous renal replacement therapy; IABP, intra-aortic balloon pump; PDA, patent ductus arteriosus; VSD, ventricular septal defect; SBP, systolic blood pressure; EF, ejection fraction; CPR, cardiopulmonary resuscitation; C-section, Cesarean section; lac, lactic acid; AFE, amniotic fluid embolism; PE, pulmonary embolism*.

Data from laboratory tests in Table 3 show that nine (90%) patients presented with normal blood urea nitrogen and creatinine at the time of ECMO initiation. Despite different etiologies, all 10 (100%) patients showed an elevation of liver enzyme indicators related to poor condition. Coagulation disorders occurred in nine (90%) patients, of which one was treated with thrombolytic therapy. Five (50%) patients had thrombocytopenia, two (20%) patients had slightly elevated bilirubin, and almost all patients had decreased albumin, reflecting the complexity of disease management in maternal patients.

**Table 3:** Laboratory tests at the time of ECMO initiation.

**Patient**	**1**	**2**	**3**	**4**	**5**	**6**	**7**	**8**	**9**	**10**	**Normal value**
GPT (U/L)	44	48	70	403	37	21	162	370	63	160	9–50
GOT (U/L)	60	247	166	2231	137	25	185	419	79	163	15–40
LDH (U/L))	515	859	884	2515	404	497	370	724	482	877	24–195
TBIL (μmol/L)	43.3	9.6	12.4	21.9	19	7.5	19	4.6	3.8	11.8	3.4–20.5
TB (g/L)	54.9	42	52.3	34.1	61.4	49.9	64.7	24.9	49.7	53.9	65–85
ALB (g/L)	28.4	28	28.2	16.3	35.5	28.4	44.6	14.9	24.7	28.3	40–55
WBC (10^9^/L)	11.5	14.3	12.8	10.8	15.7	18.1	36.3	8.8	13.8	4.2	3.5–9.5
HB (g/L)	97	100	114	89	109	75	103	64	81	87	130–175
PLT (10^9^/L)	63	94	100	139	194	159	343	13	333	83	125–350
INR	1.07	1.54	1.1	5.12	1.11	1.02	1.44	2.31	1.26	1.41	0.8–1.4
APTT (s)	40.1	111.4	28.5	>160	34.1	38.8	36.4	117.5	111	48.3	25–31.3
D-D (μg/L)	2050	5400	870	92530	4480	4820	32630	31200	6540	3680	
CR (μmol/L)	55	64	55	112	84	53	82	76	49	42	41–111
BUN (mmol/L)	2.63	3.16	3.09	7.1	4.27	3.45	6.18	3.74	5.94	2.53	3.1–8.8

*GPT, glutamic pyruvic transaminase; GOT, glutamic oxaloacetic transaminase; LDH, lactic dehydrogenase; TBIL, total bilirubin; TB, total albumin; ALB, albumin; WBC, white blood cell; HB, hemoglobin; PLT, platelets; INR, international normalized ratio; APTT, activated partial thromboplastin time; D-D, d-dimer; CR, creatinine; BUN, blood urea nitrogen*.

**Figure 1:**
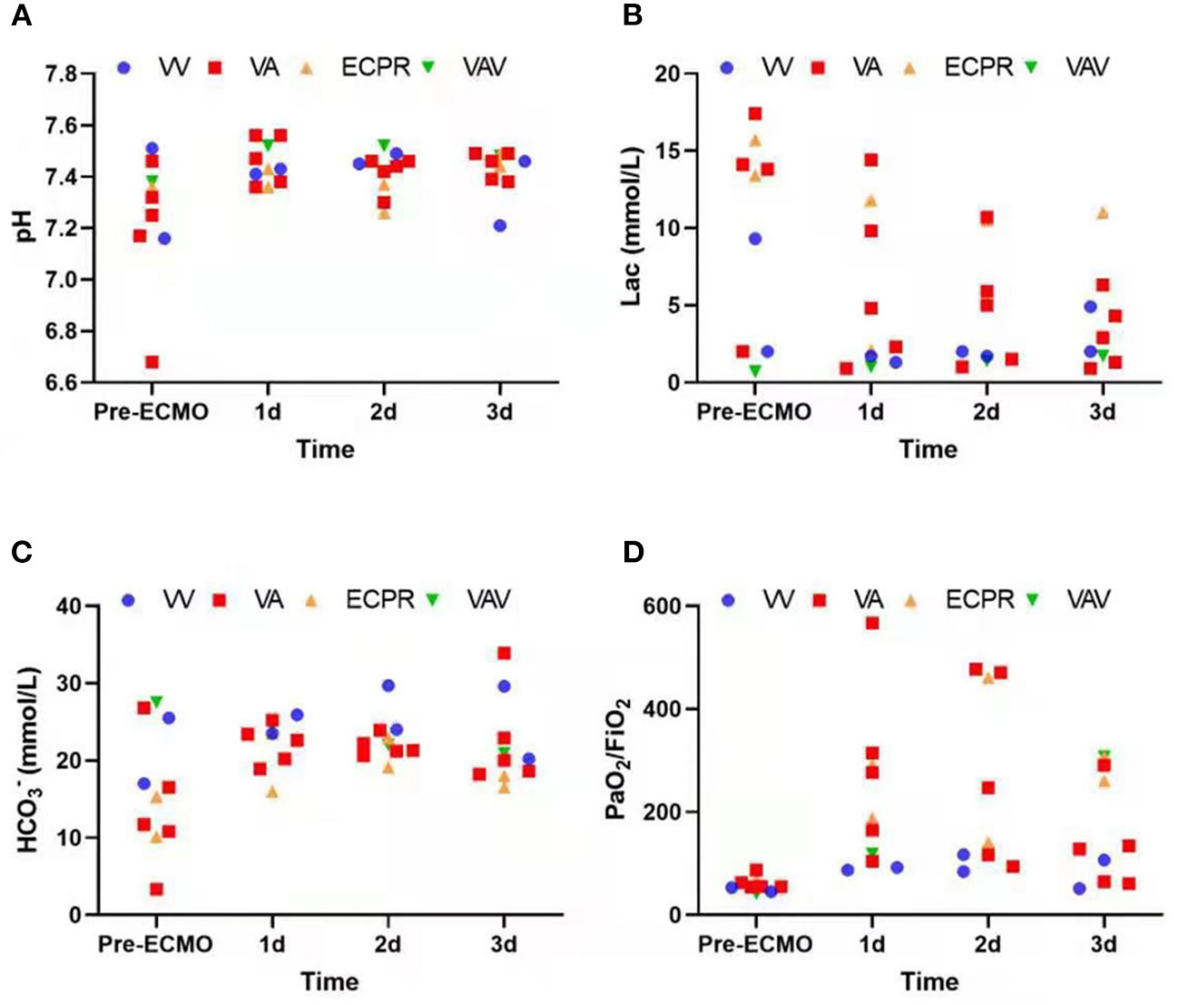
**(A–D)** Arterial blood gas trends before and during first three ECMO days.

Due to different etiologies and various treatments performed, rather than focusing on the direct relationship between ECMO and final prognosis, we instead recorded arterial blood gas at the start of ECMO and on each of the following 3 days. The data show a stabilizing trend of acid-base balance and decreasing lactic acid over 3 days following the initiation of ECMO (Figure 1). This demonstrates a promising effect of ECMO with respect to short-term stability in critical obstetric patients. Patient outcomes are listed in Table 4; seven (70%) patients survived at least 48 h after weaning from ECMO; four (40%) patients were discharged; four (40%) fetuses were discharged, while the rest were aborted or stillborn upon admission. The ECMO duration ranged from 3 to 31 days (median duration 8 days).

**Table 4:** Outcomes of ECMO and prognosis of patients.

**Patient**	**1**	**2**	**3**	**4**	**5**	**6**	**7**	**8**	**9**	**10**
Maternal survival when ECMO weaned off 48 h later	Dead	Survived	Survived	Survived	Survived	Dead	Survived	Survived	Dead	Survived
Maternal survival when discharged	Dead	Survived	Dead	Survived	Survived	Dead	Survived	Dead	Dead	Dead
Organ failure remained	Dead	None	Dead	Coma	None	Dead	None	Dead	Dead	Dead
Reason for death	Eisenmenger syndrome	N/A	Liver failure	N/A	N/A	Eisenmenger syndrome	N/A	Liver failure and sepsis	Respiratory failure and sepsis	Neurological complications after transplantation and relatives' decision
Fetal survival when discharged	Survived	Survived	Dead	Survived	Dead	Survived	Dead	Dead	Dead	Dead
Time on ECMO	14 days	7 days	8 days	11 days	8 days	8 days	4 days	3 days	7 days	31 days
Time in hospital	16 days	35 days	16 days	43 days	34 days	12 days	11 days	15 days	1 days	36 days

## DISCUSSION

We reviewed recent reports and literature of ECMO uses in the obstetric population, and found that although cases reports have increased in the last 5 years, they still remain scarce. In our study, seven of 10 patients were successfully weaned off of ECMO and survived for at least 48 h; one of these patients received a double lung transplant and survived. As reported previously by Abenhaim et al., the maternal survival to discharge rate for ECMO patients was 79.3% in published cases (13), and our study shows a similar excellent success of ECMO in the obstetric population. However, other characteristics in this population, such as gestation age, ECMO indication, neonatal survival, and long-term prognosis seem to be discriminative, which requires more attention. As these data showed, the disease of patients who needed VA-ECMO initiated seemed to progress more rapidly than those patients on VV-ECMO. Before VV-ECMO was initiated, several days of mechanical ventilation treatment may always be applied for temporary respiratory support. But what we can't ignore is that patients with slow progressing peripartum cardiomyopathy which were lacked in our study can also benefit from VA-ECMO (14). Due to sustained organ failure (i.e., liver failure and neurologic complications), only five of 10 patients survived longer than a month post-ECMO. It is worth noting that two cases of severe Eisenmenger syndrome, secondary to congenital heart disease, occurred in 2016 when we did not have access to treatments such as nitric oxide inhalation. Both patients saw improvement in conditions after ECMO support, but sadly, neither could afford heart or/and lung transplantation surgery and refused to continue treatments. Considering all factors, ECMO provided great recovery opportunities for obstetric patients, and treatment plans should be individually optimized in the future for better long-term prognosis.

Prognosis varies for pregnant women in different areas and societies due to various cultural and social security mechanisms (15). Cases of pregnant and postpartum women treated with ECMO are rarely reported in the Asian population, especially with Chinese women (16). In our study, all patients were 36 years of age or younger, which likely implies better basic organ function in these patients. Upon further analysis of laboratory tests in Table 3, it appears that liver enzyme indicators were more sensitive and potentially showed damage from ECMO initiation, whereas renal function indicators remained largely normal due to favorable basic renal function. Similar to other clinical studies (17–19), our data suggest that early application of ECMO obviously improves patient perfusion and leads to more favorable outcomes.

In obstetric patients, an excellent 73.9% neonatal survival rate was reported in a recent review, while intrauterine fetal demise occurred only in 8.9% of patients (13). In contrast, five of 10 patients in our study had an abortion or intrauterine demise prior to admission, and only 40% of infants survived until discharge. There are several objective factors that may account for this discrepancy. For instance: four patients experienced cardiopulmonary resuscitation before or during ECMO initiation; four patients were in the first trimester, which made it difficult to continue the pregnancy; and three patients were admitted to a local hospital before being transferred to our center. Two pregnant patients in their third trimester were sent to a hospital with intrauterine demise, despite receiving regular obstetric examination. Although these patients were not treated at disease onset, severe acidosis or cardiac arrest occurred upon admission. Perhaps improved obstetric examinations could reduce the probability of rapid intrauterine fetal demise outside the hospital due to maternal disease. As mentioned earlier, the two patients with severe Eisenmenger syndrome secondary to congenital heart disease struggled to maintain their pregnancies due to their condition; they were also unable to regularly participate in obstetric examinations, and pulmonary artery pressure was difficult to control upon admission. Both cases happened in 2016 we did not encounter additional obstetric patients with such severe pulmonary hypertension during the next 5 years. We now have more effective treatment measures, such as nitric oxide inhalation and heart or/and lung transplantation surgery. China is still a developing country, and many people are limited by economic conditions or low education level. Furthermore, we would like to suggest that improvement to social security mechanisms may improve the maternal death rate.

In addition to this, circumscribed retroperitoneal hematoma occurred in one patient who was 12 weeks pregnant and had fulminant myocarditis, which was considered to be a complication of ECMO. Fortunately, this patient survived after intra-abdominal hypertension for several days; after ECMO initiation, we offered to induce labor. Operating on pregnant patients requires heightened attention, regardless of gestational stage. Antoine et al. proposed a technique of ECMO cannulation using a left lateral tilt position of 15–30° during femoral cannula insertion to avoid aortocaval compression (20). We have found that careful cannula insertion with ultrasound or X-ray can help, but more clinical studies are required to confirm this finding.

Until now, no certain criteria for ECMO indication in pregnant and postpartum women had been proposed. Considering the favorable outcomes, similar criteria for ECMO in other populations seems acceptable for clinical application. Previous reports show that the most common causes leading to ECMO use in the general population are influenza-induced ARDS, pulmonary embolisms, and peripartum cardiomyopathy (14, 21, 22). However, in this study, our findings differ for pregnant and postpartum women; peripartum cardiomyopathy (80.0%) and postpartum hemorrhage (88.9%) were associated with impressive survival rate. Young patients often present with better basic conditions, and are less likely to have chronic coronary ischemia, long-term diabetes, or hypertension. Positive strategies to reverse these diseases can influence outcomes for ECMO and, therefore, the time of organ hypoperfusion can be decreased. Of note, we present two ECPR cases with unusual etiologies that contributed to cardiac arrest. One patient with induced abortion syndrome was unable to maintain effective blood pressure with continuous high-dose infusion of norepinephrine and epinephrine. The other patient with hemorrhage shock suffered cardiac arrest upon admission; rapid CPR and blood transfusion could not maintain restoration of spontaneous circulation. After ECMO initiation, an exploratory laparotomy and excision of a ruptured splenic artery aneurysm was completed. Circulation was significantly improved in both patients after ECMO initiation, and both patients survived at least 1 week post-ECMO. These cases suggest that more proactive decision-making is necessary, especially with in-hospital cardiac arrest (IHCA), considering that most etiologies resulting in cardiac arrest are reversible.

More effort needs to be focused on ECMO management for obstetric women. The complex management of anticoagulation is one of the main factors that distinguishes this population from others (23). Conventional ECMO management commonly maintains anticoagulation through heparin transfusion, which aims for 180–200 sec for activated clotting time (ACT) or 40–60 sec for activated partial thromboplastin time (APTT). Although maternal patients are often in a state of hypercoagulation, many of these patients are conversely at very high risk of bleeding from thrombocytopenia. At present, there is not a recommendation for management of anticoagulation in pregnant and postpartum women. In our clinical experience, continuous anticoagulation in patients with embolism is associated with patient outcome. When it comes to severe bleeding patients, anticoagulation can be temporarily paused; frequent monitoring of membrane function and whether there is any intracardiac thrombosis is required. ECMO can be weaned off as early as possible if conditions permit, but further studies are necessary to confirm.

CRRT has been widely used in combination with ECMO. Several studies show that CRRT decreases inflammation in an animal hemorrhage-reperfusion ECMO model (24–26). Ning Li et al. reports that CRRT alleviates the intestinal mucosal dysfunction and bacterial translocation during VV-ECMO in a porcine model (27). No significant differences have been observed in CRRT implementation in maternal ECMO. An early CRRT can help stabilize acid-base balance and liquid load management. Nevertheless, studies on long-term outcomes are lacking. Intra-aortic balloon pump (IABP), another common assistive device, is recognized for its effect on left ventricle afterload decompression (28); despite this, there is limited evidence for the effectiveness on 30-day mortality in the IABP-SHOCK II study (29). We suspect that for pregnant ECMO women, IABP can not only achieve left cardiac decompression, but also prevent a blood flow stasis (30–32). This may indirectly decrease the difficulty of anticoagulation management, and further studies are needed to confirm this.

For patients who are pregnant during ECMO, delivery of the fetus can lower aortic compression, reduce abdominal pressure, and improve maternal oxygen delivery, such that an early induction of the inviable fetus should be acceptable. In contrast, for a fetus still in gestation, individualized delivery programs must be established. The use of corticosteroids may not be clearly recommended, but is widely adapted from the literature and accelerating fetal lung maturation may improve fetal survival (33). Continuation of pregnancy is only recommended for fetuses that will survive with a follow-up short-term pregnancy. Mazzeffi et al. suggested that general obstetric indications should be retained, but more studies are expected (17). There are some limitations in our study; for example, the etiologies of cases are relatively scattered and the cases are low in number. In addition, retrospective collection of cases is prone to some bias. Thankfully, increasing numbers of ECMO studies are examining the pregnant and postpartum population, and more data will be collected and analyzed.

## Conclusion

Most pregnant women are young and have few chronic diseases. ECMO, as an auxiliary device for temporary cardiopulmonary support, can effectively stabilize the short-term condition of obstetric women and is an effective, feasible salvage treatment. For reversible disease or ECPR, aggressive strategies can be adopted to reduce the duration of patient hypoperfusion for better outcomes. Pregnant women on ECMO require more attention, especially with respect to anticoagulation and fetal management. IABP and CRRT can serve as valid adjuncts, and more studies are needed to clarify management protocols in this population.

## Data Availability Statement

The original contributions presented in the study are included in the article/supplementary material, further inquiries can be directed to the corresponding author/s.

## Ethics Statement

Written informed consent was obtained from the individual(s) for the publication of any potentially identifiable images or data included in this article.

## Author Contributions

All authors listed have made a substantial, direct, and intellectual contribution to the work and approved it for publication.

## Conflict of Interest

The authors declare that the research was conducted in the absence of any commercial or financial relationships that could be construed as a potential conflict of interest.

## Publisher's Note

All claims expressed in this article are solely those of the authors and do not necessarily represent those of their affiliated organizations, or those of the publisher, the editors and the reviewers. Any product that may be evaluated in this article, or claim that may be made by its manufacturer, is not guaranteed or endorsed by the publisher.
